# IgE-activated mast cells enhance TLR4-mediated antigen-specific CD4^+^ T cell responses

**DOI:** 10.1038/s41598-021-88956-4

**Published:** 2021-05-06

**Authors:** Binh L. Phong, Shaina J. D’Souza, Robin L. Baudier, Eric Wu, Victoria E. Immethun, David L. Bauer, James B. McLachlan

**Affiliations:** 1 Department of Microbiology and Immunology, Tulane University School of Medicine, New Orleans, LA USA; 2 Department of Epidemiology, Tulane University School of Public Health and Tropical Medicine, New Orleans, LA USA; 3 Department of Biochemistry and Molecular Biology, Tulane University School of Medicine, New Orleans, LA USA

**Keywords:** Lymphocyte activation, Mast cells, Allergy

## Abstract

Mast cells are potent mediators of allergy and asthma, yet their role in regulating adaptive immunity remains ambiguous. On the surface of mast cells, the crosslinking of IgE bound to FcεRI by a specific antigen recognized by that IgE triggers the release of immune mediators such as histamine and cytokines capable of activating other immune cells; however, little is known about the mast cell contribution to the induction of endogenous, antigen-specific CD4^+^ T cells. Here we examined the effects of specific mast cell activation in vivo on the initiation of an antigen-specific CD4^+^ T cell response. While CD4^+^ T cells were not enhanced by FcεRI stimulation alone, their activation was synergistically enhanced when FcεRI activation was combined with TLR4 stimulation. This enhanced activation was dependent on global TLR4 stimulation but appeared to be less dependent on mast cell expressed TLR4. This study provides important new evidence to support the role of mast cells as mediators of the antigen-specific adaptive immune response.

## Introduction

Mast cells (MCs) are first-line defenders of the epithelial and mucosal surfaces against allergens and invading pathogens due to their presence at the host–environment interface. They are known initiators of allergy and asthma through activation of the high-affinity IgE receptor, FcεRI, found on the cell surface^1^. Antigen (Ag) cross-linking of the IgE-bound receptors results in the release of pre-formed and de novo synthesized pro- and anti-inflammatory cytokines and mediators^2^. Aside from their well-known pathophysiological roles, MCs express a wide variety of receptors including pattern-recognition receptors (PRRs) such as the Toll like family of receptors (TLRs), which allow them to elicit specific responses to pathogens^3,4^. Although historically thought to be predominantly very early mediators of immunity, mounting evidence shows that MCs play a role in recruiting neutrophils, inducing local inflammation, and activating CD8^+^ T cells ^5–7^. Additionally, MCs are capable of priming antigen presenting cells (APCs) such as dendritic cells (DCs), which can then present Ag to T cells^8,9^. Through secreted granules and direct crosstalk, MCs also support DC migration to the draining lymph nodes and subsequent T cell priming following LPS-induced inflammation^10^. Contrastingly, studies have shown that MC gene products and cytokines can create an immunosuppressive tissue state through CD4 regulatory cell (T_reg_) function^11^. This dichotomy of playing both pro- and anti-inflammatory roles in immunity has led to the hypothesis that MCs are “tunable” based on the immunological circumstance^12^. With this early evidence showing IgE-mediated MC roles in adaptive immunity, we sought to find whether this priming could lead to an Ag-specific T cell response^13–15^.

Although the role of MCs in some aspects of adaptive immunity has been investigated, the link between MCs and endogenous Ag-specific CD4^+^ T cell immunity following immunization is less clear^12^. While it was recently shown ablating MC in the face of a potent and non-specific inflammatory response has no effect of CD4^+^ T cell activation, less is known about how direct activation of MC might affect the CD4^+^ T cell response^1,16^. Here, using an in vivo model of IgE-mediated intradermal MC activation combined with a model CD4^+^ T cell Ag, we sought to explore how highly specific MC activation might activate CD4^+^ T cells. This is relevant to human disease as it is known, in people, that IgE specific for gram positive and negative bacterial pathogens is prevalent during atopic dermatitis or bronchial asthma^17–21^. This also led us to examine how FcεRI engagement on MCs affected the Ag-specific CD4^+^ T cell response in the presence of bacterial pathogen associated molecular patterns, such as the Toll like receptor 4 (TLR4) agonist lipopolysaccharide (LPS), which might be expected to be present in these atopic patients. We found that, while specific MC activation alone was insufficient to expand endogenous Ag-specific CD4^+^ T cells, activating MC in the presence of the TLR4 ligand LPS significantly enhanced T cell activation compared to either MC activation or LPS stimulation alone. Previous studies have assessed the CD4^+^ T cell response following activation with the chemical activator compound 48/80 or vaccine induced inflammatory stimuli, both of which lack specificity for MC activation^16^. To avoid the possibility of bystander activation of other immune cells, we utilized a system where MCs were activated in a highly specific manner by crosslinking Ag-specific IgE on their cell surfaces using injection of the Ag itself. Taken together, these data provide insight into the ability of MCs to fine-tune the outcome of an adaptive T cell response based on the presence of external stimuli that are often found during infection. These results also address a fundamental gap in the MC field which has become controversial in recent years, namely that MCs may not exert fundamental effects on the adaptive immune response^22^. Understanding the ways in which MCs can act as rheostat not only increases our understanding of how MCs participate in the adaptive immune response but also allows us to exploit them for better vaccine outcomes^23^.

## Results

### In vivo IgE-mediated MC activation does not enhance the endogenous Ag-specific T cell response

**Figure 1:**
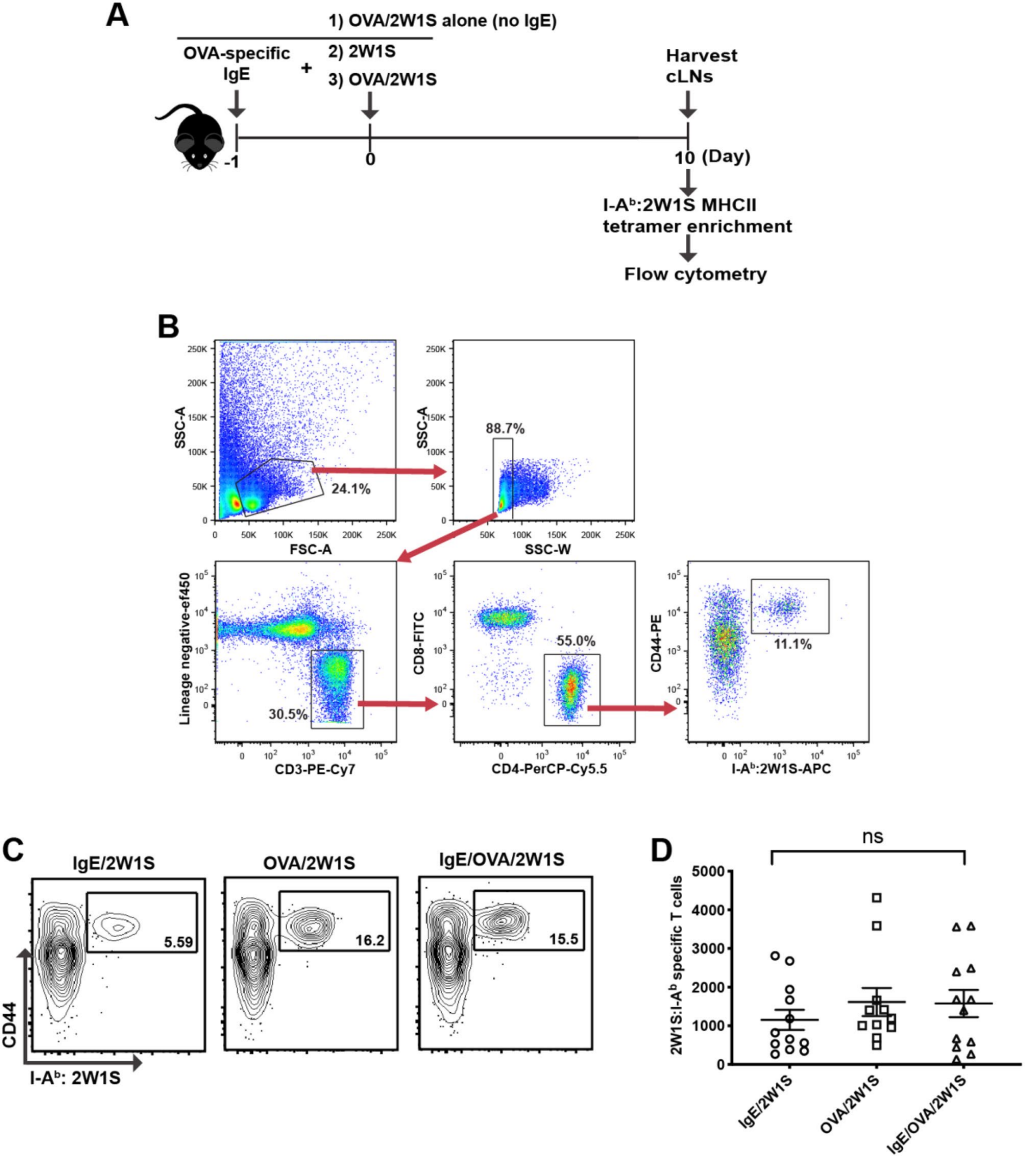
IgE-mediated mast cell activation does not result in increased numbers of endogenous antigen-specific CD4^+^ T cells. (**A**) Strategy for intradermal ear immunization to enumerate 2W1S-specific CD4^+^ T cells upon IgE/OVA-induced mast cell activation. (**B**) Gating strategy and representative flow plots of antigen-specific CD4 + T cells in the cervical draining lymph nodes (CLNs) 7 days post immunization. 2W1S-specific CD4 + T cells post enrichment were identified as lymphocytes by forward and side scatter properties and then doublets were excluded based on side scatter width (SSC-W). Singlets were stained for T cells by eliminating T cell lineage negative cells (CD11b-, CD11c-, F4/80-, CD19-), and gating on CD3ε + cell. Helper T cells were gated as CD4 + CD8α- cells. Finally, antigen experienced 2W1S specific cells were gated as CD44hi and I-Ab :2W1S-APC + . Percentages in each gate are indicated. (**C**) Representative flow plot of 2W1S-specific CD4^+^ T cells in the cervical draining lymph nodes (CLNs) 7 days post immunization for each group is shown. Percentage of 2W1S-specific CD4^+^ T cells post tetramer enrichment was indicated. (**C**) The total number of 2W1S-specific CD4^+^ T cells in the CLNs 7 days post immunization. Results are shown as mean ± SEM from 4 independent experiments *n* = 11–12 mice per group. Statistical analysis was performed using a Kruskal–Wallis test with Dunn’s correction.

Initially, we determined whether IgE-mediated MC activation promotes induction of endogenous Ag-specific CD4^+^ T cell responses. To do this C57BL/6 mice were initially sensitized intradermally by injecting IgE specific for chicken egg ovalbumin (OVA) that binds to and loads MC FcεRI in the ear skin. One day later, these sensitized MC were specifically activated by intradermally injecting OVA to cross link the IgE on the MC surface. This activation was performed in the presence of the model Ag 2W1S that is recognized by endogenous CD4 T cells as foreign in C57BL/6 mice and where we can precisely detect the presence and activation of 2W1S-specific T cells in response to the antigen (Supplemental Fig. 1). We hypothesized that MC activation combined with 2W1S would expand 2W1S-specific CD4 T cells. Control mice were injected with either OVA plus 2W1S in the absence of IgE sensitization or 2W1S alone without OVA in IgE sensitized mice to confirm that there were no bystander effects of the injection procedure (Fig. 1A). We then quantified the number of endogenous 2W1S-specific CD4^+^ T cells from injection site draining cervical lymph nodes (CLNs) seven days after injection using 2W1S MHC class II tetramer labeling and enrichment^24^. Endogenous 2W1S-specific, Ag-experienced CD4^+^ T cells were identified as CD44^+^/I-A^b^:2W1S^+^ cells (Fig. 1B). Compared to IgE sensitization alone or OVA administration alone, FcεRI crosslinking did not alter the number of 2W1S-specific T cells in either the CLNs or the spleen (Fig. 1C,D). This result suggested that specific IgE-mediated MC activation alone was not sufficient to induce Ag-specific CD4^+^ T cell immunity. Our findings did not rule out a potential role for MCs as contributors to T cell responses in the presence of other danger signals that might be expected to be present during infection. MCs express functional TLRs and stimulation via these receptors are thought to be partly responsible for protection against viral, bacterial, and parasitic infections^25^. Since MCs are thought of as “tunable” due to their ability to release specific mediators based on the types of stimuli, we next investigated whether IgE-mediated MC activation in the presence of LPS, a Toll-like Receptor 4 (TLR4) agonist, would have an effect on Ag-specific CD4^+^ T cell expansion.

### In vivo IgE-mediated MC activation in the presence of LPS leads to an increased number of endogenous Ag-specific CD4^+^ T cells

**Figure 2:**
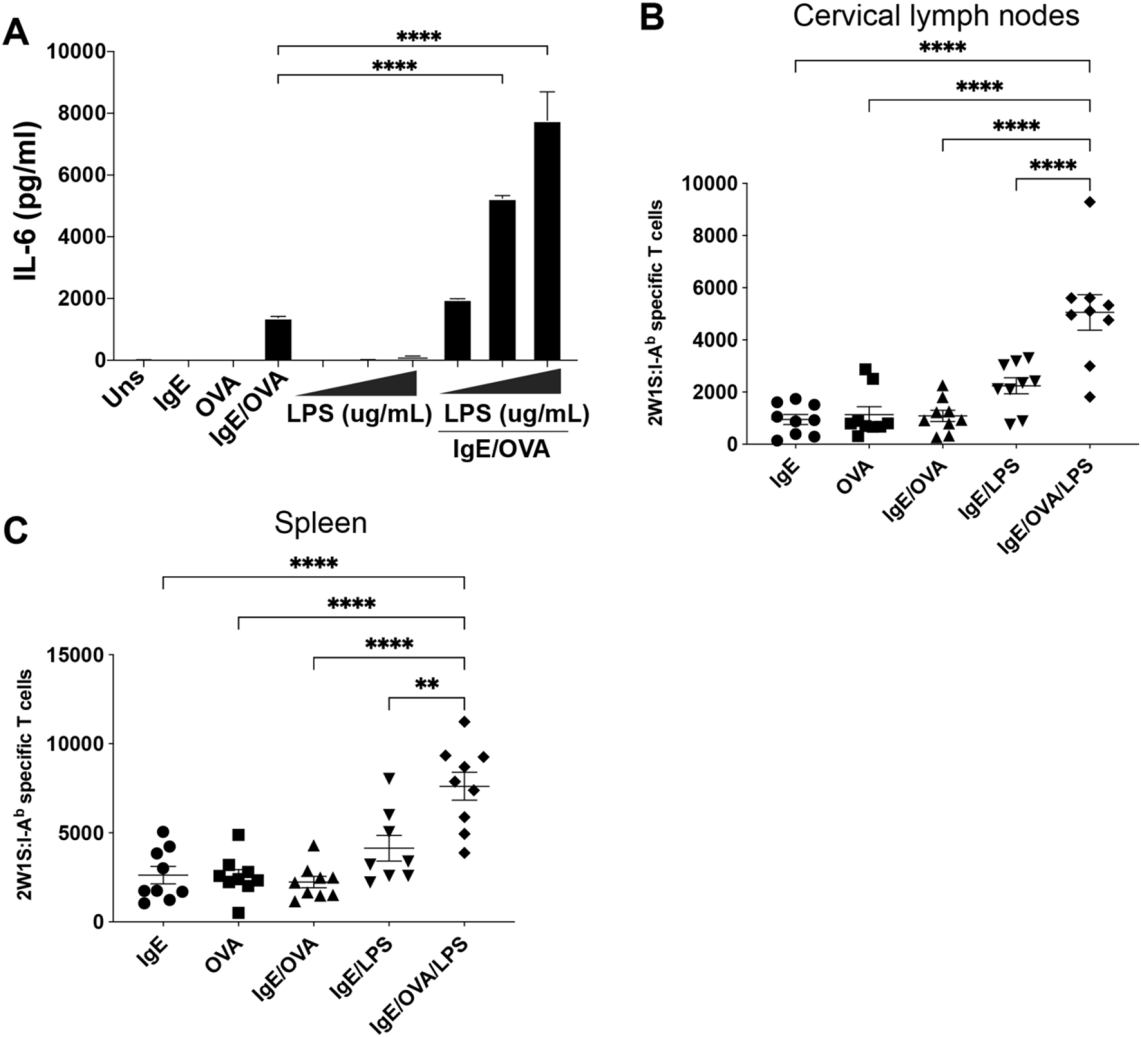
IgE/OVA and LPS co-exposure leads to expanded antigen-specific CD4^+^ T cell population. (**A**) Bone marrow-derived mast cells (BMMCs) from C57BL/6 J mice sensitized overnight with 1 μg/mL anti-OVA IgE were stimulated with 10 μg/ml OVA alone or in combination with 0.1, 1, 10 μg/ml LPS. Unsensitized BMMCs were stimulated with 0.1, 1, 10 μg/ml LPS alone. IL-6 cytokine release from supernatants was assayed by ELISA 6 h post stimulation. (**B**,**C**) C57BL/6 J mice received intradermal ear injection of 2W1S peptide and either LPS alone or IgE/OVA. The numbers of 2W1S-specific CD4^+^ T cells in the cervical draining lymph nodes (**B**) and spleens (**C**) were quantified 10 days post immunization. Results are representative of 2 independent experiments (**A**) and cumulative data from 3 independent experiments with *n* = 9 mice total (**B**,**C**) and shown as mean ± SEM. *P < 0.05 **P < 0.01, **P < 0.001, **** *P* < 0.0001. Statistical analysis was performed using one-way ANOVA with Tukey’s multiple comparisons test.

FcεRI and TLR4 have been shown to act synergistically on MC cytokine release in vitro in rodent primary and or immortalized MC lines^26^. Bone marrow-derived MCs (BMMCs) that were sensitized with OVA-specific IgE and later stimulated with a combination of OVA and LPS had a synergistic increase in IL-6 cytokine release that was not observed to the same degree with LPS exposure or IgE receptor crosslinking alone (Fig. 2A). Additional testing of the synergistic effect between IgE, OVA, and LPS was performed using a weighted least squares regression. In a saturated model with an adjusted *R*^2^ of 0.99, IL-6 expression levels were found to significantly increase with treatment with 10 μg/mL of LPS in combination with IgE by 124.0 pg/mL (*P* = 0.006), the interaction between IgE and OVA was found to increase IL-6 expression by 1372.8 pg/mL (*P* < 0.001) and the interactions between IgE, OVA and LPS of levels 0.1, 1, and 10 were found to increase IL-6 expression by 604.1 pg/mL (*P* < 0.001), 3864.3 pg/mL (*P* < 0.001) and 6274.7 pg/mL (*P* < 0.001) respectively, confirming the synergistic effect of combined treatments. (Full equation in Materials and Methods). Further, when we tested the ability of IgE, OVA, and LPS to activate BMMCs derived from TLR4 knockout mouse bone marrow, we found that the synergistic effect was lost in the knockout cells compared to WT BMMC (Supplemental Fig. 2) confirming that TLR4 signaling essential for the combined effect on MC IL-6 release. Murine MC IL-6 and histamine secretion along with engagement of the OX40/OX40L signaling pathway has been shown to inhibit Treg functions leading to higher activation of effector CD4^+^ and CD8^+^ T cells^27–29^. This increase of IL-6 secretion in vitro led us to hypothesize that co-stimulation via FcεRI and TLR4 in vivo could lead to an increased Ag-specific CD4^+^ T cell response that is dependent on TLR4 signaling.

Using the previously described model, we first sensitized mouse ears with intradermally injected OVA-specific IgE. The next day, mice were injected intradermally in the same location with 2W1S and either OVA (to stimulate sensitized MCs) or LPS, or a combination of OVA and LPS. As observed previously (Fig. 1C,D), MC activation alone did not promote further expansion of 2W1S-specific T cells in the draining lymph nodes and spleen (Fig. 2B,C). There was a slight increase in the number of 2W1S-specific T cells in mice receiving LPS, consistent with LPS as an immunogenic agent^30–32^. However, when MCs were activated in the presence of LPS, there was a significant increase in the number of 2W1S-specific T cells recovered in both the CLNs and spleens from those same mice compared to either treatment alone (Fig. 2B,C). Testing of the synergistic effect between IgE, OVA, and LPS on T cell counts were performed using a linear regression. In a model with an adjusted *R*^2^ of 0.64, the combined treatment with IgE and LPS was found to increase T cell counts by an estimated 1289 total Ag-specific cells (*P* = 0.02) and the interaction between IgE, OVA, and LPS were found to increase T-cell counts by an estimated 2675 total Ag-specific cells (*P* = 0.001), confirming the synergistic effect of combined treatments. (Full equation in Materials and Methods). These results suggest that IgE-mediated MC activation in the presence of LPS leads to a synergistic expansion of Ag-specific T cells. One possible explanation for this increase in T cells is that LPS has been shown to enhance MC degranulation in allergic asthmatic mice leading to increased cytokine release that may activate T cells^33^. There is also evidence that LPS can suppress the IgE-mediated MC responses, and potentially modulating subsequent T cell responses, through decreased surface expression of FcεRI and associated increase in IL-10^34^. Furthermore, our results did not confirm whether LPS acted in costimulation with FcεRI crosslinking directly on MCs or through another cell type. Therefore, we further investigated whether MCs themselves were required for an elevated Ag-specific T cell response.

### MCs are required for an elevated Ag-specific T cell response

**Figure 3:**
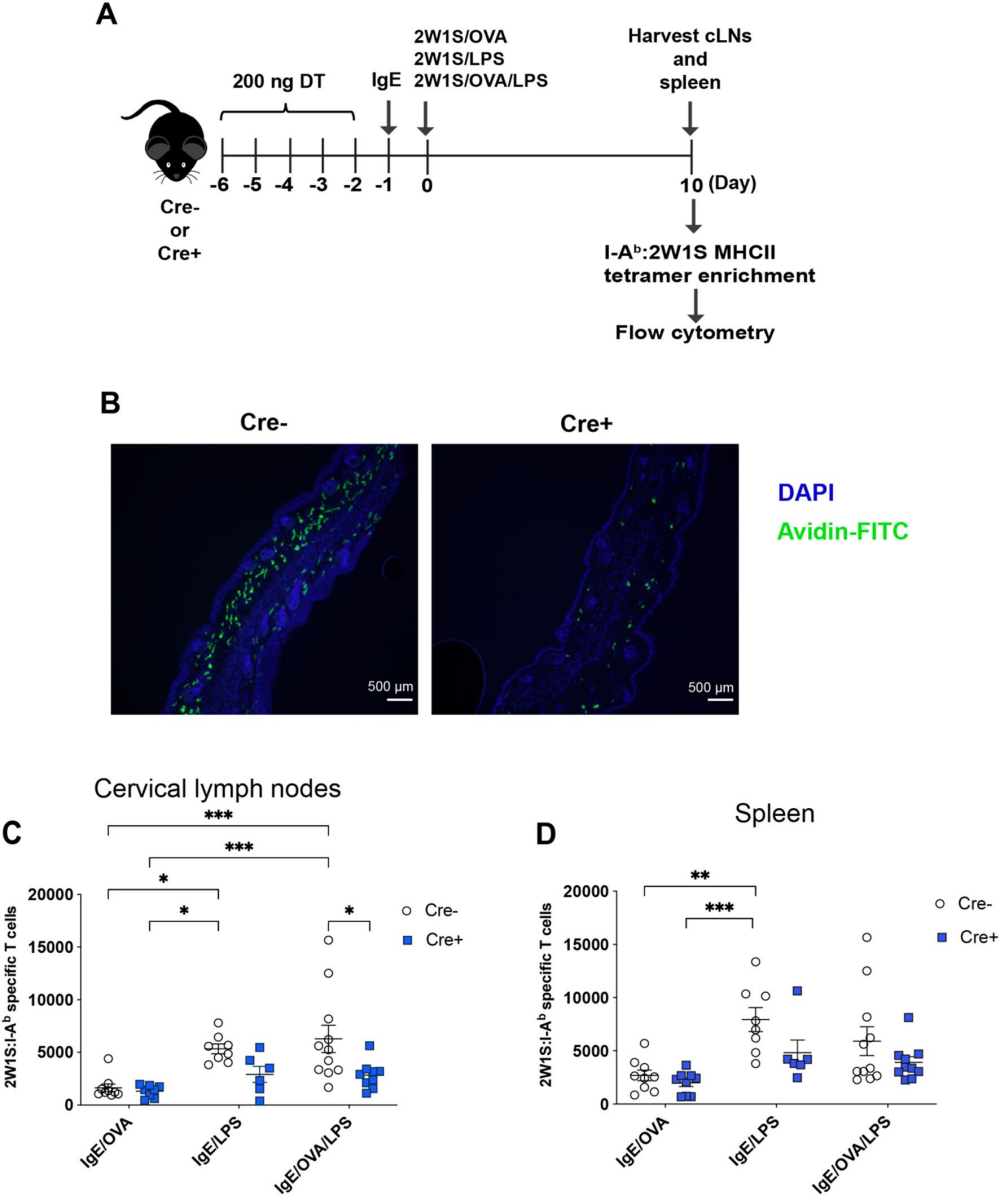
Mast cells are required for expansion of antigen-specific CD4^+^ T cells upon IgE mediated activation and LPS exposure. (**A**) Mast cell depletion and intradermal ear immunization strategy to quantify the number of 2W1S-specific CD4^+^ T cells in Mcpt5-Cre + (Cre +) mice and littermates (Cre −) upon 2W1S peptide, IgE/OVA, and LPS stimulation. (**B**) Immunofluorescent images of mast cells visualized by Avidin-FITC in ear tissue sections from Cre + and Cre − mice to show mast cell reduction in Cre + mice one day post last DT injection taken at 10 × objective. The numbers of 2W1S-specific CD4^+^ T cells within the cervical draining lymph nodes (**C**) and spleen (**D**) of mast cell-depleted (Cre +) mice and littermate controls (Cre −) 10 days post intradermal ear immunization. Results are cumulative data from 3 independent experiments *n* = 6–11 mice total and shown as mean ± SEM. *P < 0.05 **P < 0.01, **P < 0.001, **** *P* < 0.0001. Statistical analysis was performed using one-way ANOVA with Tukey’s multiple comparisons test (**C**) or a Kruskal–Wallis test with Dunn’s correction (**D**). Scale bar, 500 µm.

To determine if MCs directly contributed to endogenous Ag-specific T cell response, we utilized the *Mcpt5-Cre x iDTR* mice where MCs can be conditionally ablated by administration of diphtheria toxin (DT)^35–37^. The *Mcpt5-Cre x iDTR* mice were chosen because of their intact c-Kit expression, retention of mucosal MCs, and similar basophil numbers to WT mice^38–41^. Mice were injected with 200 ng of DT intravenously for 5 days to ensure connective tissue MC ablation prior to antigen delivery and MC activation (Fig. 3A). Immunofluorescence (IF) microscopy images of ear tissue sections taken on the day of IgE sensitization, one day post the last DT injection, showed reduced MC numbers in Cre + mice compared to Cre- littermate controls (Fig. 3B). Ablation of MCs significantly diminished the 2W1S-specific CD4^+^ T cell response to LPS plus IgE crosslinking (Fig. 3C). This difference was less obvious in the spleen (Fig. 3D). These results indicated that MCs, when sensitized and activated via their IgE receptors, were important contributors to amplify the inflammatory signal provided by LPS, leading to expansion of endogenous Ag-specific T cells in the skin-draining lymph nodes.

### TLR4 mediates the costimulatory effect of LPS and IgE-mediated MC activation to enhance endogenous Ag-specific T cell response

**Figure 4:**
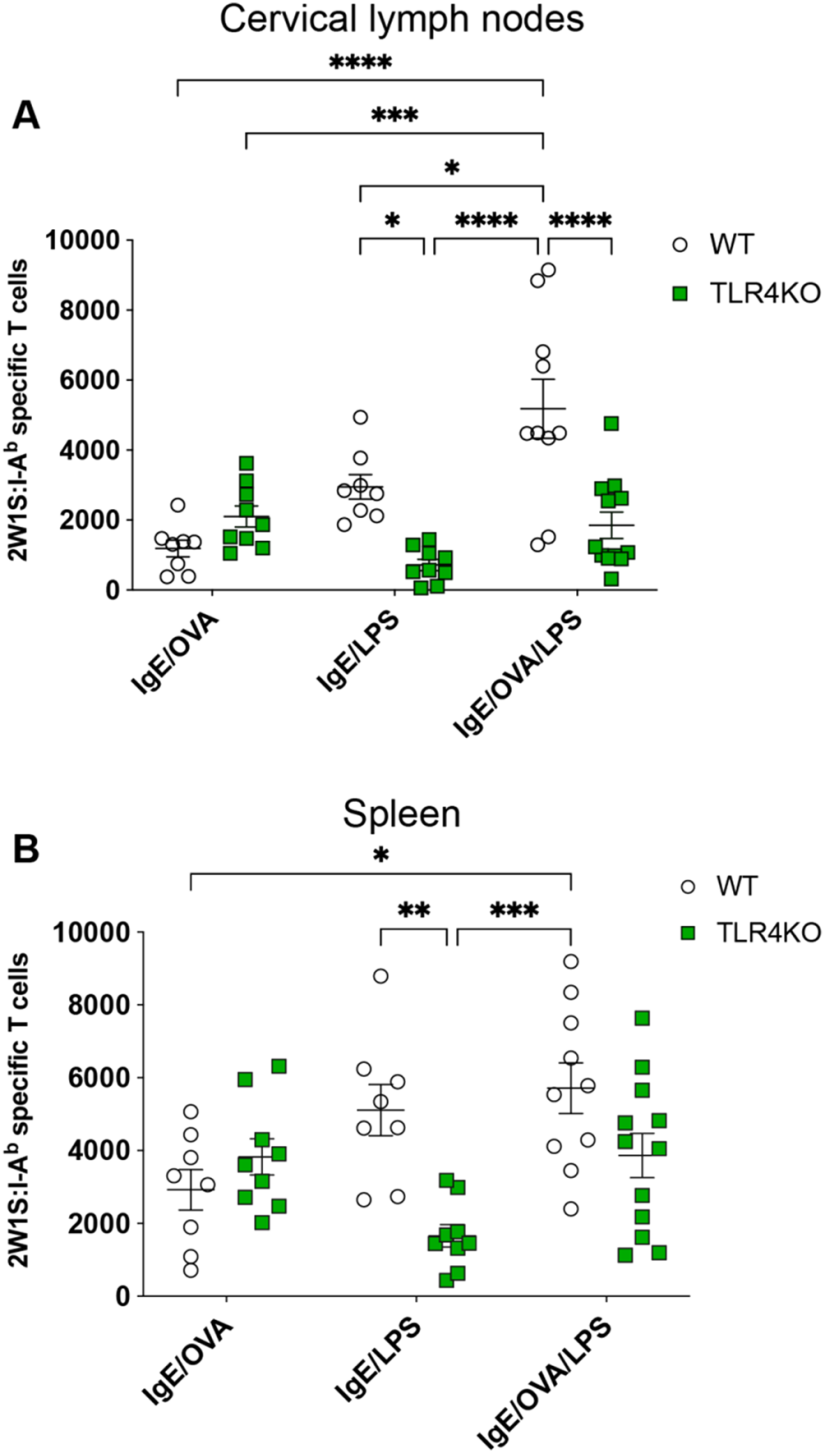
TLR4 activation by LPS is required for enhanced antigen-specific CD4^+^ T cell response upon IgE/OVA-induced mast cell activation. (**A**,**B**) WT or TLR4-deficient mice were immunized with 2W1S peptide, IgE/OVA, LPS, or a combination of IgE/OVA and LPS. Cervical draining lymph nodes (**A**) and spleens (**B**) were analyzed 10 days post immunization for the numbers of 2W1S-specific CD4^+^ T cells. Results shown are cumulative data from 3 independent experiments *n* = 8–12 mice and shown as mean ± SEM. *P < 0.05 **P < 0.01, **P < 0.001, **** *P* < 0.0001. Statistical analysis was performed using one-way ANOVA with Tukey’s multiple comparisons test.

To ensure the effects we observed with LPS costimulation were mediated by its receptor TLR4, we assessed the number of 2W1S-specific CD4^+^ T cells in *Tlr4*-/- mice subjected to IgE/OVA and LPS costimulation^42^. Consistent with LPS stimulating the adaptive immune response via TLR4, there was a significant reduction in 2W1S-specific T cells in TLR4-deficient mice treated with LPS compared to wild-type controls in the draining LN and spleen (Fig. 4A,B)^43^. Furthermore, when MCs were specifically activated in the presence of LPS, the increased expansion of 2W1S-specific T cells normally detected in the draining LN of wild-type mice was significantly abrogated in TLR4 knockout mice (Fig. 4A) demonstrating that TLR4 was essential for the MC amplifying effect on CD4^+^ T cells. A similar trend was observed in the spleen (Fig. 4B). Our findings suggest that the specific adaptive immune response generated by IgE-mediated MC activation is likely dependent on the presence of a TLR agonist, perhaps only LPS binding to TLR4.

### MC TLR4 expression is not not responsible for the entirety of the enhanced endogenous Ag-specific CD4^+^ T cell response

**Figure 5:**
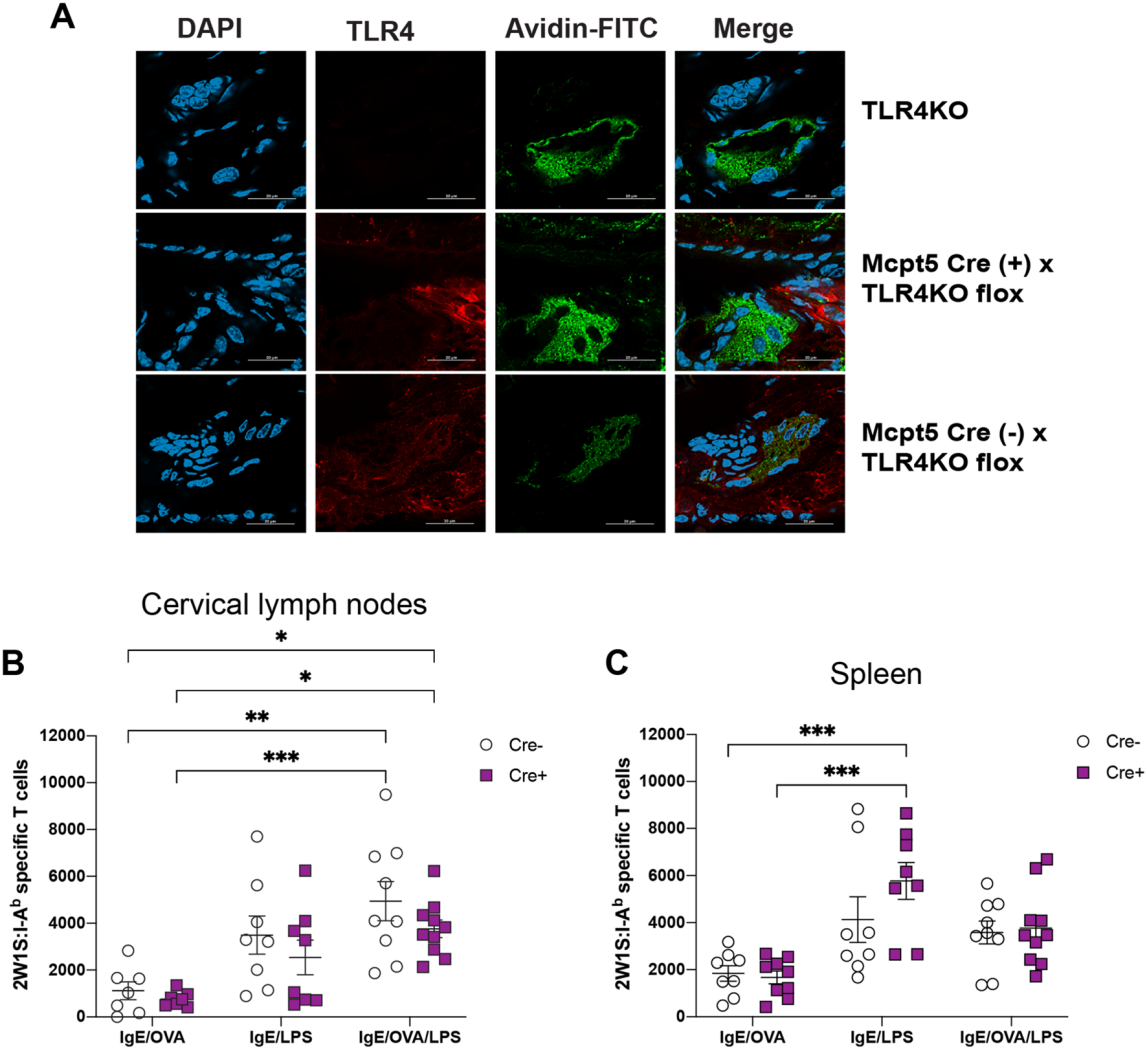
TLR4 expression on mast cells is not entirely responsible for the IgE/OVA- and LPS-mediated expansion of antigen-specific CD4 + T cells. (**A**) Tissue ear sections from TLR4-deficient mice (top), *Mcpt5* Cre ( −) x *Tlr4KO* flox (middle), and *Mcpt* Cre ( +) x *Tlr4KO* flox (bottom) were imaged using confocal microscopy (see “Materials and methods” section). Red (PE) indicates TLR4 stain, green (FITC) indicates MC-granule-specific avidin stain, and DAPI (blue) indicates cell nuclei. Image shows that Cre ( +) MCs lack TLR4 expression in comparison to Cre ( −) MCs. (**B**,**C**) Mast cell-specific TLR4-deficient mice (Cre +) and littermate controls (Cre −) were immunized intradermally in the ears with 2W1S peptide and the indicated combination of IgE, OVA, and LPS. Cervical draining lymph nodes (**B**) and spleens (**C**) were analyzed 10 days later for the numbers of 2W1S-specific CD4^+^ T cells. Results are cumulative data from 3 independent experiments with *n* = 7–10 mice total and shown as mean ± SEM. Scale bar, 25 µm. *P < 0.05 **P < 0.01, ***P < 0.001, **** *P* < 0.0001. Statistical analysis was performed using one-way ANOVA with Tukey’s multiple comparisons test.

It has been shown in multiple studies that MCs express a variety of TLRs, including TLR4 in both rodents and humans^44–48^. Our findings thus far have shown that specific MC activation through its IgE receptor promoted an Ag-specific T cell response in mice concurrently exposed to LPS; however, it was unclear whether this enhancement was due to direct costimulation of FcεRI and TLR4 on MCs leading to upregulated MC mediator release, or due to an LPS bystander effect on activated MCs. To determine whether LPS targets MC TLR4 specifically, we generated a novel mouse strain by crossing the *Mcpt5-Cre* with *Tlr4-flox* mice where TLR4 expression is deleted only in connective tissue MCs. Immunofluorescence microscopy from ear tissue staining for TLR4 and MCs confirmed the absence of TLR4 expression on dermal MCs (Fig. 5A).

Using the previous immunization strategy, we detected comparable 2W1S-specific T cell expansion whether or not TLR4 was expressed by MCs in the skin when LPS was administered in the immunization, regardless of whether MC were specifically activated (Fig. 5B,C). This expansion was similar in both the draining CLN and the spleens. Collectively, our results showed that while global TLR4 expression and activation is required for full T cell expansion, TLR4 on MCs themselves may not be as important for this expansion, although we cannot completely rule out a role for MC expressed TLR4 in our system. Here, MCs may serve as an amplifier of the signal delivered by LPS to other cell types involved in shaping the magnitude of an Ag-specific T cell response while possibly also contributing some TLR4-derived signal to the response. Our studies, for the first time, provided direct in vivo evidence that MCs can regulate the magnitude of an adaptive T cell outcome by sensing and responding to a combination of signals within in local environment.

## Discussion

There has been increasing evidence to support a role for MCs in shaping the adaptive immune response, but the field has recently been more contentious in this regard. Some early studies, including our own, showed that MCs are essential mediators of bacterial clearance and can elicit both innate and adaptive immune responses, predominantly mediated via the actions of the cytokine TNF-a^9,49–54^. Other studies, using a variety of MC depletion or deficiency models, have shown that MCs play a role in eliciting immunity against viral infections^55–58^. Many of the early studies relied on c-kit deficient mice (either W-sash or W/W^v^ mice) which potentially demonstrate off target immune effects that may make interpretation for the role of MCs complicated^59–63^. More recent studies have used targeted MC ablation models where MCs can be specifically deleted without affecting other cell types^16,38^. These studies have shown more modest roles, or even no function, for MCs in eliciting immunity and in particular, have shown that MC activators like compound 48/80 are less specific for MCs than originally observed. We sought to understand a more precise role for MCs in regulating a T cell response in the absence of these potentially confounding factors. To do this we investigated whether IgE-mediated stimulation, a more specific and physiological MC activation than previously explored, could affect the endogenous, Ag-specific CD4^+^ T cell response. In agreement with more recent studies, we found that specific MC activation alone exhibited no effect on the T cell response; however, when combined with the TLR4 agonist LPS, MCs were important amplifiers of the T cell response. This outcome was most clear in the MC activation site-draining cervical lymph nodes and was less apparent in the spleens of those same mice, a difference that has been shown before^64^. This is possibly due to the concentration of antigen specific T cells in the draining lymph nodes where the differences would be expected to be most pronounced. The spleen, as a more distal tissue, is more likely to reflect T cells that escape the draining lymph nodes and enter the circulation. Our results provide further evidence to support the emerging view of MCs as an orchestrator rather than the main driver in the induction of the T cell response. We found a clear synergistic effect for LPS combined with IgE mediated activation in wild type mice; however, that difference was less clear in the MC ablation model where the synergistic effect was lost, even though MC were clearly required to activate T cells. One explanation for this discrepancy is the potential effect DT may have on the littermate control mice in addition to the Mcpt5-cre DTR mice. While DTR engineered mouse models have been valuable in studies of immunity and disease states, DT administration to deplete specific cell types is not immunologically inert. For example, while studying initiation and breakdown of tolerance in the lung, Chapman et al. found that intra-tracheal administration of DT induced inflammation in both wild type and transgenic mice^65^. Similarly, in a pulmonary fungal infection model, local and systemic DT administration in CD11c-DTR mice led to fatal fulminant myocarditis that may be due to a radio resistant, non-hematopoietic cell type^66^. We speculate that the potential DT-induced inflammation when coupled with a strong stimulatory signal like LPS (IgE/LPS group) results in an enhanced antigen-specific T cell response that is not further elevated by IgE receptor crosslinking. Nevertheless, our data show that the synergistic increase in antigen-specific T cell response requires mast cells and possibly another cell type expressing TLR4.

Physiologically, these findings have a variety of implications in human disease, particularly with respect to atopic disease. It is known that cases of atopic dermatitis and allergic asthma are diseases sometimes associated with, and exacerbated by, bacterial infection^17,19^. In these circumstances, one would expect to find both IgE activation of MCs combined with bacterial products such as LPS. Our findings provide mechanistic and pathological insight into why pathology is increased in these cases. Additionally, it is known that MCs are important mediators of bacterial clearance, it is possible that IgE mediated activation of MCs combined with the presence of LPS evolved to enhance bacterial specific adaptive immunity and resulting bacterial clearance. While it is clear that TLR4 is required for this enhanced response, we also find that TLR4 expression on MCs themselves play less clear of a role and we cannot completely rule out the possible contribution of TLR4 espressed on MCs in our model system. While multiple studies have shown TLR4 expression on MCs, a more recent study found that MCs may express no pattern recognition receptors, implying that other cells expressing TLR4 might synergistically cooperate with MCs to amplify the T cell response^44,48,67^. Although principally characterized in relation to the innate immune system, studies have shown that TLR4 activation can lead to increased T cell-mediated immune responses and different T helper responses, making the receptor a highly sought-after target for vaccine adjuvants^68–72^. Notably, CD4 T cells themselves express TLR4 on the surface^73^, so it is possible that the LPS effect we observe also involves T cells directly responding to TLR4 ligation in addition to their response to MC derived factors. The role MCs play in TLR4 activation in the adaptive immune system, apart from the pro-inflammatory cytokines released when stimulated on the MC surface, is unclear^74^. We show here that in vivo, MCs appear to cooperate specifically with TLR4 ligation to induce greater expansion of Ag-specific CD4^+^ T cells. Future studies will explore other potential TLR4 expressing cells that might mediate this effect.

It is known that MC products can regulate DC maturation, migration, and subsequent T cell phenotypes and MCs can form synapses with DCs to facilitate antigen transfer and subsequent T cell activation^9,10,75–78^. DCs express high levels of TLR4, so it is likely that LPS acts on DCs which in turn receive boosting signals from MCs to subsequently activate CD4^+^ T cells. Indeed, TLR4 activation of DCs is a crucial pathway connecting the innate and adaptive immune response since TLR4 stimulation induces DC maturation by upregulation of costimulatory molecules and production of pro-inflammatory cytokines^79,80^. Combined, our results show that exposure to bacterial pathogens in an allergic setting like IgE-mediated MC activation can lead to more a robust and synergistic Ag-specific CD4^+^ T cell response, an outcome that could be both beneficial in defense against pathogens and problematic in the exacerbation of allergic diseases. The ability to respond to both harmless allergens and pathogenic signals in a stimuli-specific manner places MCs in a unique position to be both helpful and detrimental to the host’s response to the external environment. Understanding the multifunctional role of MCs, both as an effector and a regulator in the innate and adaptive responses, will lead to better strategies to combat diseases where allergic predispositions are prevalent.

## Materials and methods

### Mice

Mice used included C57BL/6J (Charles River, #027), *Tlr4* KO (Jackson Laboratory, #029015), *Tlr4 floxed (*Jackson Laboratory, #024872), iDTR and *Mcpt5-Cre* + (provided by Axel Roers, University of Technology, Dresden, Germany). All mice were used between 6 and 10 weeks. For mast cell depletion in Mcpt5-Cre + x iDTR mice, 8-week old mice were given 200 ng Diphtheria Toxin (DT, Unnicked, from *Corynebacterium diphtheriae,* List Biological Laboratories) per mouse intravenously once a day for 5 consecutive days prior to intradermal ear immunization. Mcpt5-Cre (- x iDTR mice were used as littermate controls. Control mice were also injected with DT to control for off target effects caused by DT. To generate mast cell-specific TLR4-deficient mice, Mcpt5-Cre + mice were crossed with *Tlr4-floxed mice.* Cre-non-expressing animals were used as littermate controls. Animal breeding was conducted in accordance with recommendations from the Guide for the Care and Use of Laboratory Animals of the National Institutes of Health. All experimental procedures were approved and performed in compliance with the guidelines established by Tulane University School of Medicine’s Institutional Animal Care and Use Committee. This study was also carried out in compliance with the ARRIVE guidelines.

### Antibodies

The antibodies used in flow cytometry and immunofluorescence microscopy were CD4 (RM4.5, BD PharMingen), CD11b (M1/70, Tonbo Biosciences), CD11c (N418, Tonbo Biosciences), F4/80 Antigen (BM8.1, Tonbo Biosciences), B220 (RA3-6B2, BD PharMingen), CD3e (145-2C11, Tonbo Biosciences), CD8 (53–6.7, BioLegend), CD44 (IM7, BioLegend), FcεRI (MAR-1, eBioscience), c-Kit (2B8, eBioscience), avidin (Av-FITC, BioLegend), TLR4 (HTA125, eBioscience), and IgG (H + L) Secondary Antibody (A-21434).

### Bone marrow-derived MC culture and in vitro assays

Bone marrow-derived mast cells (BMMCs) were generated by culturing bone marrow from adult C57BL/6J or TLR4 KO mice for 4–6 weeks in DMEM supplemented with 10% Bovine Calf Serum, 10 mM L-Glutamine, 5 mM Sodium Pyruvate, 1% Pen-Strep, 55 μM beta-mercaptoethanol, and IL-3-conditioned media from WEHI-3 cells (provided by Sarah Gaffen, University of Pittsburgh, USA). Cells were used when at least 90% positive for c-Kit and FcεRI by flow cytometry.

### BMMC stimulation and IL-6 cytokine measurement

BMMCs were sensitized with 1 μg/mL anti-OVA IgE (E-C1, Chondrex) overnight in complete media without IL-3. Cells were then stimulated with 10 μg/mL OVA (EndoFit Ovalbumin, InvivoGen) unless otherwise indicated, alone or together with 0.1, 1, 10 μg/mL of LPS-SN (LPS from *S. minnesota R595,* InvivoGen) in IL-3-free media for 6 h. Supernatants were assayed for murine IL-6 by ELISA (BioLegend).

### Intradermal ear immunizations

Anti-OVA IgE (E-C1, Chondrex), OVA (EndoFit Ovalbumin, InvivoGen), recombinant mouse serum albumin (rMSA, Albumin Bioscience), LPS-SN (LPS from *S. minnesota R595,* InvivoGen), Diphtheria Toxin (DT, Unnicked, from *Corynebacterium diphtheriae,* List Biological Laboratories), and 2W1S peptide (sequence: EAWGALANWAVDSA, GenScript Biotech) were used. All reagents were dissolved in DPBS (-Ca, -Mg). For intradermal immunization, mice were injected in both ears with 50 ng anti-OVA IgE diluted in 1 mg/mL rMSA in PBS or buffer alone. Then, 24 h later, mouse ears were injected intradermally with 15 mg 2W1S peptide and, based on experimental groups, a combination of 10 mg OVA, or 100 ng LPS as indicated. Cervical draining lymph nodes (CLNs) and spleens were harvested 7 or 10 days post immunization.

### Generation of single cell preparations, 2W1S-specific T cell enrichment, and flow cytometry

Single cell suspensions from CLNs and spleens were obtained by homogenization through a 100 mm nylon mesh filter in cold sorter buffer (1 × phosphate buffered saline, 2% newborn calf serum, 1 mM EDTA, and 0.1% sodium azide) and resuspended in FC block (Clone 2.4G2 SFM supernatant + 2% mouse serum, 2% rat serum, 0.1% NaN3) prior to incubation with 10 nM I-A^b^ :2W1S-APC MHC-II tetramer in the dark for one hour at room temperature. 2W1S-specific T cells were enriched by magnetic separation with anti-APC magnetic beads (Miltenyi) as described previously (Moon et al., 2009). Briefly, cell suspension was incubated with 25 μL of anti-APC microbeads for 30 min on ice in the dark. Cells were then washed, resuspended in cold sorter buffer, and applied to Miltenyi LS Columns placed on a quadroMACS magnet over a nylon mesh. The columns were rinsed two more times with cold sorter buffer. 2W1S tetramer-bound cells were released from the columns by addition of cold sorter buffer to the columns off the magnet.

To identify the population of 2W1S-specific CD4 + T cells, the enriched cell suspension above were stained with lineage negative antibodies (eFluor450-labeled CD11b, CD11c, F4/80, and CD19), PE-Cy7-CD3, PerCP-Cy5.5-CD4, FITC-CD8, and PE-CD44 antibodies. Cells were collected on a LSR Fortessa (Beckton-Dickson) or a FACSCelesta Flow Cytomer (Beckton-Dickson). Data were analyzed using FlowJo software (TreeStar). Tetramer-positive cells were gated as “Dump” negative (CD11b-, CD11c-, F4/80-, CD19-), CD3ε + , CD4 + , CD8α-, CD44^hi^, and I-A^b^ :2W1S-APC + . To quantify the numbers of antigen-specific CD4 + T cells, 5 μL of unstained cells was removed from a known volume of cell suspension post tetramer enrichment and added to 200 μL of Accucheck counting beads (Invitrogen) at a known concentration. The total number of tetramer positive cells was calculated using the following formula:

$$ \left( {\begin{array}{*{20}c} {Total\;cell\;number } \\ {in\;a\;sample} \\ \end{array} } \right) = \left( {\frac{cell\;count}{{bead\;count}}} \right) \left( {\begin{array}{*{20}c} {bead} \\ {stock\;concentration} \\ \end{array} } \right) \left( {\frac{bead\;volume}{{cell\;volume}}} \right) \left( {\begin{array}{*{20}c} {total} \\ {sample\;volume} \\ \end{array} } \right) $$

The number of antigen-specific T cells was calculated by multiplying total cell number with the percentage of tetramer-positive cells.

### Fluorescent immunohistochemistry

To confirm mast cell depletion in Mcpt5-Cre + x iDTR mice, Avidin-FITC staining for mast cells was performed on ear sections harvested from mice 1 day after the last DT injection. 10 μm tissue sections were fixed for 20 min with 4% paraformaldehyde in PBS and permeabilized with 0.1% Triton-X 100 (Sigma-Aldrich) in PBS for 5 min at room temperature. Specimens were blocked with 0.5% bovine serum albumin (BSA, Grainger) in PBS for 15 min at room temperature. Ear tissues were then stained with 25 ng/mL Avidin-FITC (BioLegend) and DAPI (2 drops in 1.5 mL volume) (NucBlue Fixed Cell ReadyProbes Reagent, Thermo Fisher Scientific) in 0.5% BSA in PBS for 30 min at room temperature in the dark. Slides were mounted with Prolong Gold Antifade Mountant (Thermo Fisher Scientific) for 24 h at room temperature in the dark prior to imaging on Nikon Eclipse TE300 microscope (Nikon) with a 10 × objective.

For confocal imaging, ear tissue sections were fixed and permeabilized as described above. Specimens were blocked with 5% normal goat serum (NGS, Thermo Fisher Scientific) in PBS for 1 h at room temperature. Tissues were then stained with TLR4 (HTA125, eBioscience) for two hours in the dark. Then, tissues were stained with Secondary Antibody (A-21434), avidin (Av-FITC, BioLegend), and DAPI (2 drops in 1.5 mL volume) (NucBlue Fixed Cell ReadyProbes Reagent, Thermo Fisher Scientific) in 5% NGS in PBS for 1 h at room temperature in the dark. Slides were mounted as described previously. Images were acquired with a Nikon Ti2 inverted microscope connected to a Nikon A1plus camera. The objective used was a Nikon 60 × CFI Plan Apo Lambda oil immersion lens with a 1.4 NA. Acquisition software used was Nikon NIS-Elements Confocal 4.60. Images were taken at 22 °C. Post-capture operations that were done include minor LUT adjustments to reduce background (the same LUT settings were applied to all images) and 2D automatic deconvolution through Nikon’s Enhanced Resolution software module in the Elements software.

### Data analysis and statistics

All statistical analysis was performed using Prism (GraphPad) software v. 9 or R v.3.6.1. All groups were assessed for normality and if distribution was found to be normal, one-way ANOVA with Tukey’s or Holm-Šídák's multiple comparisons tests were used to determine statistical significance and p values, as appropriate. If distribution was found to be non-normal, we assessed significance using Kruskal–Wallis test with Dunn’s correction for multiple comparisons Outliers were removed using QuickCalcs outlier calculator (GraphPad) on a per group basis (i.e. CLN constituted a group). For regression analyses, linear regression was used when homoscedasticity assumptions were met and weighted least squares regression was used when they were not. IL-6 pg/mL was predicted by weighted least squares regression using the following equation:

$$ \begin{aligned} & {\text{IL}}6 = { }7.7{ }{-}{ }1.9{ }\left( {{\text{IgE}}} \right){ }{-}{ }5.4\left( {{\text{OVA}}} \right){ }{-}{ }5.8\left( {{\text{IgE*LPS }}0.01} \right){ }{-}{ }2.3{ }\left( {{\text{IgE*LPS }}0.1} \right) \\ & \quad \quad \quad \quad \quad + \;{ }9.3{ }\left( {{\text{IgE*LPS }}1} \right) + { }124.0{ }\left( {{\text{IgE*LPS }}10} \right) + { }1372.8\left( {\text{IgE*OVA}} \right) \\ & \quad \quad \quad \quad \quad + { }\;110.5\left( {{\text{IgE*OVA*LPS }}0.01} \right) + 604.1\left( {{\text{Ige*OVA*LPS }}0.1} \right) \\ & \quad \quad \quad \quad \quad + \;3864.3\left( {{\text{IgE*OVA*LPS }}1} \right) + 6274.7\left( {{\text{IgE*OVA*LPS }}10} \right){ } \\ \end{aligned} $$

where the intercept (7.7) represented the media alone group.

T cell counts were predicted by linear regression using the following equation:

$$ Tcells = 949.1 + 185.6 \left( {OVA} \right) - 48.1\left( {IgE*OVA} \right) + 1289.38\left( {IgE*LPS} \right) + 2675.6\left( {IgE*OVA*LPS} \right) $$

where the intercept (949.1) represented IgE treatment alone.

## Supplementary Information

Supplementary Information

## Abbreviations

MC: Mast cells
TLR: Toll like receptor
Ag: Antigen
OVA: Ovalbumin
