# Supplementary material for: IgE-activated mast cells enhance TLR4-mediated antigen-specific CD4+ T cell responses
Source: Sci Rep. 2021 May 6;11:9686. doi: 10.1038/s41598-021-88956-4 (PMC8102524; doi:10.1038/s41598-021-88956-4)
Supplement: Supplementary file 1 — Supplementary Information [file 41598_2021_88956_MOESM1_ESM.docx]

**
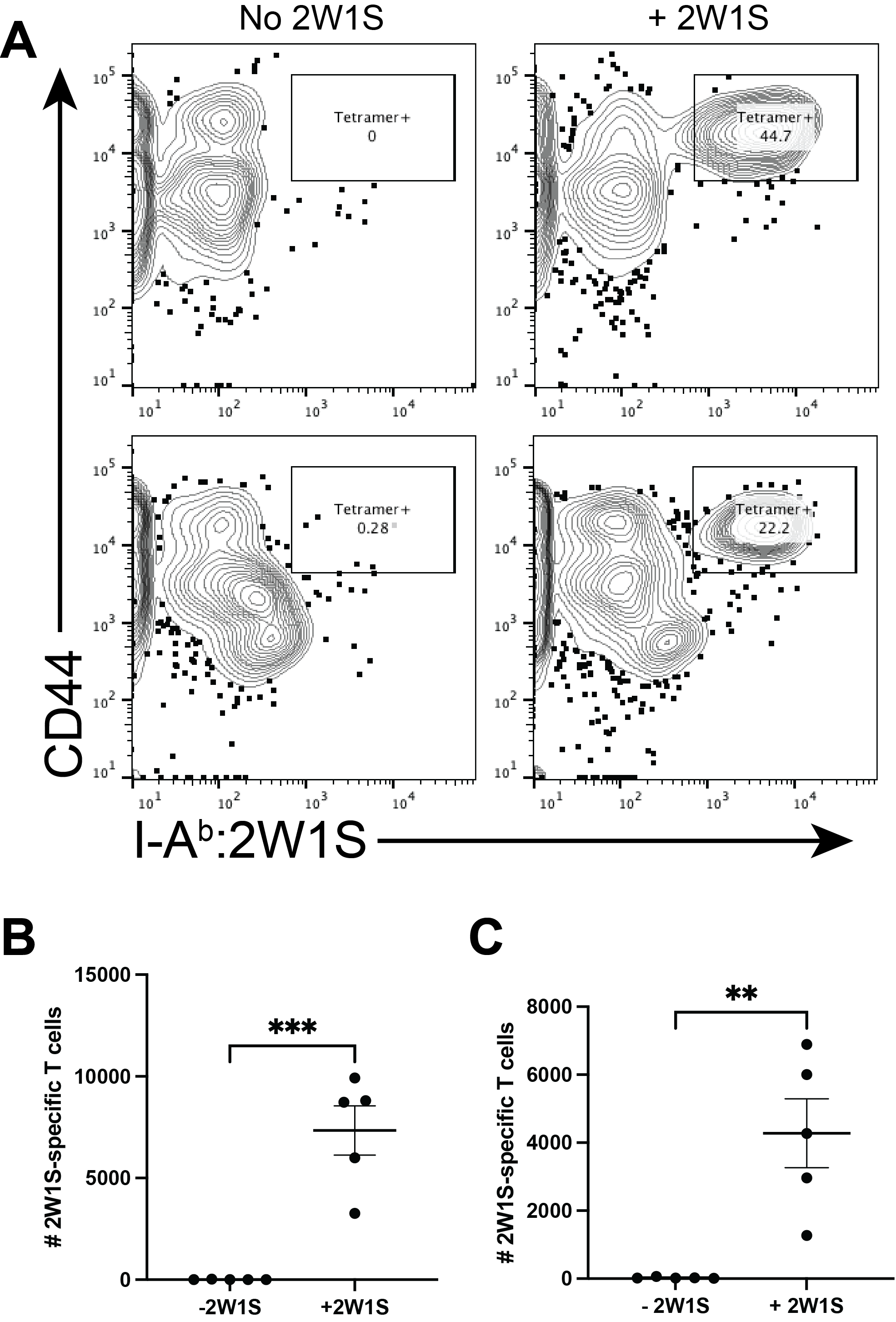
Supplemental Figure 1. IgE-mediated mast cell activation without additional antigenic 2W1S peptide does not result in an increased number of endogenous antigen-specific CD4+ T cells.**  WT mice were sensitized intradermally with OVA-specific IgE and were then immunized with OVA/LPS with or without 2W1S peptide. (A) Representative flow plots for each group are shown for CLN (top) or spleen (bottom) either eith or without 2W1S added. Total number of 2W1S-specific CD4+ T cells in the (B) cervical draining lymph nodes or (C) spleens 10 days post immunization were analyzed using flow cytometry. Data is shown as mean +/- SEM. Statistical analysis was performed using Student’s t-test. ***P* <0.01, ****P* <0.001

**Supplemental Figure 2. IL-6 release after IgE/OVA and LPS coexposure *in vitro*is lost in BMMCs derived from TLR4-deficient mice**. Bone marrow-derived mast cells (BMMCs) from C57BL/6J and TLR4-deficient mice sensitized overnight with 1 μg/mL anti-OVA IgE were stimulated with 10, 50, 100 μg/ml OVA alone or with 10 μg/ml OVA in combination with 0.1, 1, 10 μg/ml LPS. Unsensitized BMMCs were stimulated with 10 μg/ml of LPS alone. IL-6 cytokine release from supernatants was assayed by ELISA 6 hours post stimulation. Results are representative of 2 independent experiments (in triplicate) and shown as mean ± SEM.  **** P <0.0001. Statistical analysis was performed using two-way ANOVA with Šídák's multiple comparisons test.
